# Our Experience of Deep Sclerectomy at a Tertiary Center in the United Kingdom Over 14 Years

**DOI:** 10.7759/cureus.43366

**Published:** 2023-08-12

**Authors:** James Richardson-May, Rawdha Alnuaimi, Ahmed Elbably, Lawrence Walker, Suresh Thulasidharan, Richard Dacombe, Aby Jacob

**Affiliations:** 1 Ophthalmology, University Hospital Southampton National Health Services (NHS) Foundation Trust, Southampton, GBR; 2 Clinical Outcomes, University Hospital Southampton National Health Services (NHS) Foundation Trust, Southampton, GBR

**Keywords:** glaucoma, cataract, elevated intraocular pressure, non-penetrating glaucoma surgery, glaucoma surgery, deep sclerectomy

## Abstract

Background

Deep sclerectomy (DS) is a non-penetrating surgical procedure for glaucoma, reducing the resistance to aqueous outflow and lowering intraocular pressure while maintaining a physiological barrier between the anterior chamber and the sub-scleral space. This offers a lower complication profile than penetrating procedures, though with less intraocular pressure (IOP) reduction.

Methods

We retrospectively reviewed the electronic record for all DS undertaken at our hospital (a tertiary care center) over 14 years, collecting data on demographics, diagnosis, IOP, visual acuity, complications, medications, and further procedures required.

Results

Eighty eyes of 69 patients underwent DS, with a mean follow-up period of 53.5 months. The mean pre-operative IOP was 23.55 mmHg (range 11-52, standard deviation 8.46); the mean final IOP was 13.61 mmHg (range 5-35, SD 4.73), with a mean reduction of 42.21%. The mean change in glaucoma medications was -1.64. 78.40% experienced a reduction in glaucoma treatment. Post-operatively, 43.80% had no complications; this improved to 85.0% when numerical hypotony and raised IOP without visual sequelae were excluded. Further procedures required included Nd:YAG goniopuncture (10%), bleb needling (13.75%) or revision (7.5%), iridectomy (3.75%), goniosynechiolysis (1.25%), and autologous blood injection (1.25%). Two eyes were converted to trabeculectomy peri-operatively, with seven overall (8.75%) requiring trabeculectomy over the course of follow-up. 3.75% underwent glaucoma drainage device implantation, and 3.75% underwent cyclodiode laser.

Conclusion

We have found DS to be a safe, effective procedure for selected patients where trabeculectomy has a high likelihood of failure or where a higher IOP can be tolerated.

## Introduction

Deep sclerectomy (DS) is a non-penetrating filtering surgery (NPFS) for glaucoma, aiming to lower intraocular pressure (IOP) without entering the anterior chamber (AC). This offers certain benefits outside of penetrating surgery, such as trabeculectomy, by maintaining a physiological barrier between the AC and sub-scleral space. It allows the filtration of aqueous humor through the created trabeculo-Descemet’s window (TDW) into an intrascleral reservoir while preserving the AC’s immune-privileged state. The indications for DS include most forms of open-angle glaucoma, including primary (POAG), pseudoexfoliative and pigmentary, glaucoma associated with myopia, aphakia or pseudophakia, open-angle uveitic glaucoma, normal tension, and steroid-induced glaucoma [1].

DS has been found to be useful in eyes that require concurrent cataract surgery or have a history of uveitis; these eyes are prone to inflammation post-operatively, which increases the risk of failure of traditional filtering surgery such as trabeculectomy [2]. The DS procedure maintains a physiological barrier between the AC and sub-scleral space [3], which helps to limit the incited inflammation. We undertook a retrospective review of all DS procedures at our hospital over a 14-year period as part of an internal audit to assess our surgical outcomes and to compare standalone DS with that combined with cataract surgery amongst our cohort of patients. We hope to add to an expanding pool of literature on this procedure, in addition to a literature review examining the role of DS in general.

## Materials and methods

We retrospectively reviewed the electronic record for all eyes that underwent DS at our hospital (a tertiary care center in the United Kingdom) from May 2007 to September 2021 as part of an internal audit of our practice. We collected data on demographics, diagnosis, IOP, best corrected visual acuity (VA), complications, medications, and further procedures required. We included all patients who underwent the procedure during this timeframe. Data was collated on Microsoft Excel (Redmond, USA), and analysis was undertaken on both Microsoft Excel and GraphPad Prism software. Unpaired t tests were used to assess the statistical significance of subgroups where relevant.

Surgical technique

DS was undertaken in our hospital by a single surgeon (AJ). It was primarily indicated in forms of open-angle glaucoma, particularly those where traditional penetrating surgery such as trabeculectomy was felt to have a high risk of failure, such as in combined cases with cataract surgery and uveitic glaucoma. Concurrent phacoemulsification and intraocular lens insertion were performed in cases with visually significant cataracts. In cases with angle closure, the iridocorneal angle had to be sufficiently open in the region of the TDW. This was confirmed preoperatively with gonioscopy. In those who were undergoing phacoemulsification surgery alongside DS with a history of angle closure, following phacoemulsification and insertion of the intraocular lens, the angle was checked intraoperatively to ensure there was significant space for DS. If not, viscosynechiolysis was undertaken to open the angle further.

To perform the procedure, a conjunctival peritomy is undertaken superiorly, following which mitomycin-C (MMC) is applied subconjunctivally at a dose of 0.2 mg/ml for 3 minutes using a polyvinyl acetate sponge. A superficial scleral flap, between one-third and one-half scleral thickness, is formed, following which a deeper flap is dissected. This is advanced anteriorly, beyond the scleral spur, and the Schlemm canal is deroofed. This deeper flap is then excised to form the trabeculo-Descemet’s window (TDW), a thin membrane between the AC and sub-scleral lake that allows the filtering of aqueous. An implant (ESNOPER, AJL Ophthalmic) or a dispersive viscoelastic (Viscoat, Alcon) can be used to maintain this space, with our preference being viscoelastic. The superficial flap and conjunctiva are sutured with interrupted 10-0 vicryl sutures, and subconjunctival steroids and antibiotics (e.g., dexamethasone and cefuroxime) are given. The patient is commenced on topical steroids, and antibiotic drops post-operatively.

## Results

Eighty eyes of 69 patients underwent DS. The mean age was 71.60 years (range 16 to 95 years, standard deviation 16.09). 48.80% were male, and 51.20% were female. The mean follow-up period was 53.50 months (range 1-66 months, SD 44.95). Table 1 describes ocular co-pathology. 

**Table 1:** Ocular co-pathology in all eyes undergoing deep sclerectomy.

Co-pathology	N
Documented multiple drop intolerance	1
Trauma	
Subluxed crystalline lens	1
Cornea	
Previous penetrating keratoplasty	7
Previous endothelial keratoplasty	3
Microbial keratitis (bacterial)	1
Microbial keratitis (viral)	1
Microbial keratitis (acanthamoeba)	1
Conjunctival intraepithelial neoplasia	1
Band keratopathy	1
Previous glaucoma procedures	
Selective laser trabeculoplasty	2
Argon laser trabeculoplasty	2
Cyclodiode laser	1
Uveitis	
Fuch’s heterochromic iridocyclitis	2
Punctate inner choroidopathy	1
Retina	
Retinitis pigmentosa	1
Retinal vein occlusion	2
Dry age-related macular degeneration	3
Wet age-related macular degeneration	1
Previous vitrectomy	3
Non-proliferative diabetic retinopathy	5
Proliferative diabetic retinopathy	1

The primary glaucoma diagnosis was POAG in 68.75%. Secondary open-angle glaucoma was the next most common, including corticosteroid-induced (8.75%), surgical (5%), traumatic, pseudoexfoliative, and uveitic (2.5% each). 1.25% had a combination of uveitic and corticosteroid-induced glaucoma, and 2.5% had a combination of pigment dispersion and corticosteroid-induced glaucoma. Normal-tension glaucoma was the primary diagnosis in 1.25%. 5% had a form of angle closure, including primary angle closure (2.5%), primary angle closure glaucoma (1.25%), and secondary angle closure from previous surgery (1.25%); in these cases, the angle was open in the region that surgery was undertaken, or the DS was combined with cataract surgery to open the angle before DS.

DS was combined with phacoemulsification in 57.5% of cases. 65% received antimetabolite during surgery; this was mitomycin C (MMC) in all documented cases. Most procedures were undertaken without a spacer to maintain the sub-scleral space (86.25%), though dispersive viscoelastic (VisCoat, Alcon) was utilized to maintain this space instead. Two eyes (2.5%) were converted to trabeculectomy during the procedure.

Pre-operative IOP was available for 76 eyes, with a mean IOP of 23.55±8.46 mmHg. Mean first post-operative IOP (the first IOP reading recorded following the procedure) was 11.73±7.03 mmHg, and the mean final IOP was 13.61±4.73 mmHg, with a mean change in IOP (n=76) of -9.96 mmHg.

**Figure 1:**
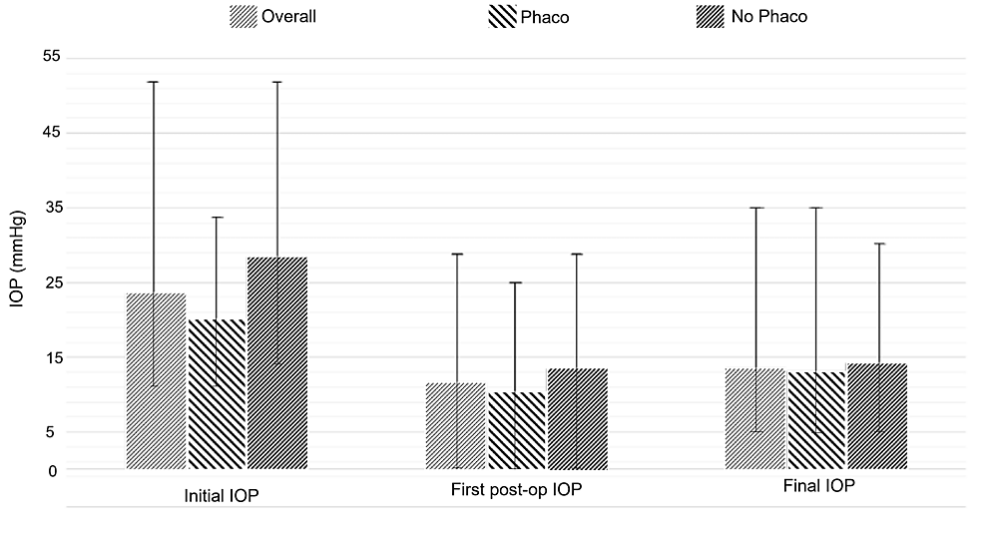
Comparison of mean initial IOP, first post-operative IOP, and final IOP in patients undergoing DS. Values are mmHg. Error bars demonstrate range.

**Figure 2:**
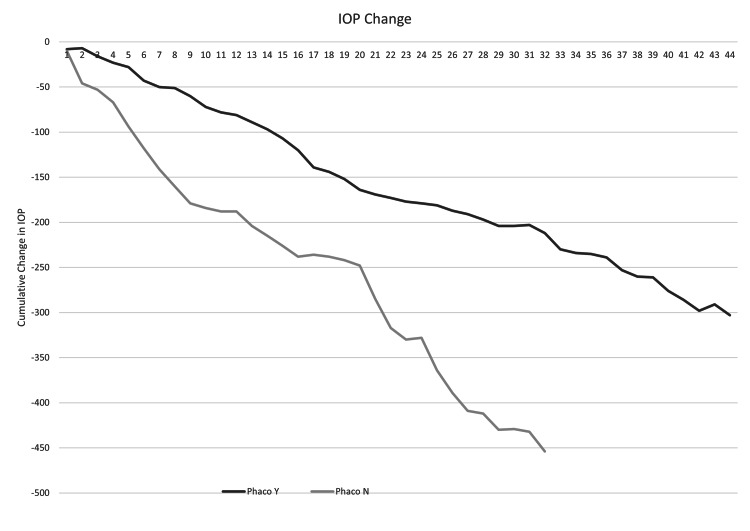
Comparison of change in IOP in eyes with concurrent phacoemulsification (“Phaco Y”) and without (“Phaco N”)

In the 34 eyes with no concurrent phacoemulsification, the mean pre-operative IOP was 28.44±9.88 mmHg. The mean first post-operative IOP was 13.50±8.14 mmHg, and the final IOP was 14.21±4.84 mmHg, with a mean change of -14.19 mmHg. In the 46 eyes that had concurrent phacoemulsification, the mean pre-operative IOP was 20.00±4.75 mmHg. The first post-operative IOP was 10.41±5.74 mmHg, the final IOP was 13.17±4.60 mmHg, and the mean change was -6.89 mmHg (Figures 1, 2). There was no significant difference in final IOP between these groups (p=0.14).

Pre-operatively, the median number of glaucoma medications was 2.5±1.0. Median post-operative use was 0±2, with a mean change of -1.64 glaucoma medications; 78.40% of eyes experienced a reduction in glaucoma treatment.

VA improved or was maintained at 66.0%. Peri-operative complications were rare, with 83.75% having no complications. Post-operatively, 43.80% had no complications; this improved to 85.0% when numerical hypotony and transient raised IOP without visual sequelae were excluded (Table 2).

**Table 2:** Complications in all patients undergoing deep sclerectomy, both peri- and post-operative.

Complication	N	Percentage (%)
Peri-operative		
Hyphaema	3	3.75
Posterior capsular rupture	1	1.25
Scleral flap tear	2	2.5
Descemet’s membrane rupture	1	1.25
Sclerectomy perforation	1	1.25
Corneal perforation (from traction suture)	1	1.25
Iris prolapse through sclerectomy (replaced)	1	1.25
Choroidal exposure	1	1.25
Conversion to trabeculectomy	2	2.5
Post-operative		
Hypotony		
Numerical alone	14	17.5
Maculopathy	1	1.25
Choroidal effusion	6	7.5
Shallow anterior chamber	1	1.25
Other		
Choroidal effusion with normal IOP	1	1.25
Bleb leak	5	6.25
Raised IOP (>21 mmHg)	20	25.0
Iris incarceration	4	5.0
Anterior uveitis	1	1.25
Corneal epithelial defect	1	1.25
Hyphaema	1	1.25
Flap haemorrhage	1	1.25

Further procedures required (Table 3) included Nd:YAG goniopuncture (10%), bleb needling (13.75%) or revision (7.5%), iridectomy (3.75%), goniosynechiolysis (1.25%), and autologous blood injection (1.25%). Two patients were converted to trabeculectomy peri-operatively, with seven overall (8.75%) requiring trabeculectomy over the course of follow-up. 3.75% underwent glaucoma drainage device (GDD) implantation, and 3.75% required a cyclodiode laser.

**Table 3:** Further procedures are required post-deep sclerectomy. SLT: selective laser trabeculoplasty. IOL: intraocular lens. YAG: Neodynium-doped yttrium aluminum garnet laser.

Procedure	Eyes (n)	Procedures (n)	Percentage of eyes undergoing procedure (%)	Notes
Glaucoma				
YAG goniopuncture	8	8	10	-
Bleb needling +/- antimetabolite	11	15	13.75	-
Bleb revision	6	8	7.5	-
Iridectomy	3	3	3.75	-
Goniosynechiolysis	1	2	1.25	-
Trabeculectomy	7	7	8.75	Includes 2 converted peri-operatively
Glaucoma drainage device (tube)	3	4	3.75	1: Ahmed 1: Baerveldt 2: not documented
Cyclodiode	3	6	3.75	-
SLT	3	4	3.75	-
Autologous blood injection	1	1	1.25	-
Cataract				
Phacoemulsification and IOL	2	2	2.5	-
YAG posterior capsulotomy	9	10	11.25	-
Cornea				
Penetrating keratoplasty	1	4	1.25	-
Corneal biopsy	1	1	1.25	-
Other				
Pars plana vitrectomy	1	3	1.25	-
Evisceration	2	2	2.5	-

Thirty eyes experienced hypotony (IOP ≤6 mmHg) at some point over the course of the follow-up. Of these, one developed hypotony maculopathy and required bleb revision. Six patients developed choroidal effusions; one subsequently underwent resuturing of the bleb, with the rest resolving without surgical intervention. One eye had shallowing of the anterior chamber due to overfiltration, which again resolved without surgery. Twenty (73.3%) had no structural or visual complications as a result of this (“numerical hypotony”). The primary diagnosis in this subgroup was POAG in 59.10%, pseudoexfoliative glaucoma (4.50%), pigmentary dispersion with corticosteroid-induced glaucoma (9.10%), uveitic (4.50%), corticosteroid-induced (13.60%), and surgically induced open-angle glaucoma (9.10%). Table 4 lists the ocular co-morbidities of this group. Fourteen patients had concurrent use of MMC, with eight receiving no anti-metabolite. In this group of eyes with post-operative hypotony, the mean pre-operative IOP was 24.30±8.90 mmHg. The first post-operative IOP was lower than the overall DS group, at 6.50±6.50 mmHg (p<0.0001), with a final IOP of 11.60±4.10 mmHg.

**Table 4:** Ocular co-morbidities in eyes with hypotony, and those with raised IOP post-operatively.

Co-morbidity	N
Hypotony	
Cornea	
Band keratopathy	1
Previous penetrating keratoplasty	3
Previous endothelial keratoplasty	1
Microbial keratitis (viral)	1
Microbial keratitis (acanthamoeba)	1
Uveitis	
Punctate inner choroidopathy	1
Previous glaucoma procedures	
Previous argon laser trabeculoplasty	2
Retinal	
Non-proliferative diabetic retinopathy	2
Proliferative diabetic retinopathy	1
Previous pars plana vitrectomy	1
Raised IOP (>21 mmHg)	
Cornea	
Previous penetrating keratoplasty	2
Previous endothelial keratoplasty	2
Uveitis	
Anterior uveitis	1
Previous glaucoma procedures	
Previous cyclodiode	1
Previous selective laser trabeculoplasty	1
Previous argon laser trabeculoplasty	1
Retinal	
Non-proliferative diabetic retinopathy	1
Retinal vein occlusion	1
Previous pars plana vitrectomy	2
Other	
Trauma (subluxed crystalline lens)	1

Conversely, 19 eyes were found to have raised IOP (>21 mmHg) during their postoperative course. The main glaucoma diagnosis was POAG (40%); PXFG, uveitic with corticosteroid-induced glaucoma, PACG, secondary angle closure glaucoma, pigment dispersion with corticosteroid-induced glaucoma, and secondary open-angle glaucoma following surgery (5% each); and uveitic, traumatic, or corticosteroid-induced alone (10% each). Ocular co-morbidities are listed in Table 4. Thirteen eyes had concurrent antimetabolites (11 received MMC, 2 were not recorded). The mean pre-operative IOP was 27.70±9.04 mmHg. The first post-operative IOP was 15.30±8.57 mmHg, with a final IOP of 13.90±5.98. Further procedures were common in this group. 35% underwent Nd:YAG goniopuncture; 40% underwent bleb needling; 15% had bleb revision surgery, and 5% each underwent iridectomy and autologous blood injection to the bleb. 5% required cyclo-diode later in their course, with 10% undergoing GDD surgery and 20% needing trabeculectomy. 15% of eyes had selective laser trabeculoplasty (SLT).

We determined failure rates based on an IOP equal to or lower than 6 mmHg or equal to or higher than 21 mmHg at the final follow-up. In addition, a drop in VA to NPL or PL or the need for further glaucoma surgery (excluding YAG goniopuncture). Based on these criteria, 26 patients (32.5%) failed over the course of follow-up, with three IOP too high and six being too low. Three patients lost vision to PL or worse. Twenty-three patients in total required further glaucoma surgery over the course of follow-up; of these, 17 could be considered a qualified success, with their final IOP meeting success criteria. As such, our complete success rate was 67.5%, with a complete and qualified success rate of 88.8%.

## Discussion

DS is an NPFS with the potential to avoid some complications of penetrating surgery, including hypotony, hyphaema, endophthalmitis, and cataracts [1]. It is useful in eyes with concurrent cataracts or uveitis, where penetrating surgery can incite inflammation, increasing the risk of trabeculectomy failure [2]. The procedure reduces aqueous resistance and lowers IOP while maintaining a physiological barrier between the AC and sub-scleral space [3].

In DS surgery, a conjunctival peritomy is followed by the application of an antimetabolite agent such as Mitomycin-C (MMC); we used a dose of 0.2 mg/ml on a polyvinyl acetate sponge and applied it for 3 minutes. A 1/3-1/2-thick superficial scleral flap is dissected, followed by a deep, almost full-thickness flap. This is advanced beyond the scleral spur, de-roofing Schlemm’s canal. The juxta-canalicular tissue is removed, and the deeper flap is excised. This forms a thin filtering membrane (the TDW) between the AC and sub-scleral reservoir of aqueous, with the formation of a bleb. Spacers or dispersive viscoelastic can be used to maintain the space between TDW and the scleral flap [1,4]. It can also be combined with viscocanaloplasty [5]. The superficial scleral flap and conjunctiva are closed with interrupted 10-0 vicryl sutures. Postoperatively, if IOP remains higher than expected, the TDW can be perforated using an Nd:YAG laser to perform goniopuncture, forming a direct fistula between the AC and bleb; this has been estimated to be required in 8.9% of patients [6]. Spacers may reduce the incidence of fibrosis and may increase the number of outflow scleral vessels. This can improve outcomes and IOP reduction [7].

In a review of 105 eyes who underwent DS, Roy and Mermoud found favorable results, with few complications over 10 years, a mean IOP of 12.20±4.70 mmHg, and a 77.6% success rate [1]. DS has been reported to have a steep learning curve [8]. This may limit its use among glaucoma specialists, particularly those who do not receive instruction in the procedure over the course of their training. Inadvertent puncture of the TDW effectively converts the DS into a trabeculectomy, a more familiar procedure for glaucoma specialists that has similar outcomes to a “classic” trabeculectomy [9]. Two patients in our study were converted to trabeculectomy peri-operatively; these eyes had good outcomes, with IOP of 12 mmHg and 9 mmHg.

Compared to trabeculectomy, DS offers fewer complications but a higher overall IOP compared to penetrating surgery. Cheng et al. undertook a systematic review to assess the efficacy of NPFS (both DS and viscocanaloplasty) in POAG. They found that trabeculectomy was more effective at lowering IOP, with trabeculectomy achieving a reduction of 45.6% in IOP compared to 35.2% in the NPFS group over two years. Augmentation with MMC improved IOP-lowering, as did the use of a spacer. They found fewer postoperative complications in the NPFS group [10]. A 2014 Cochrane review also compared NPFS with trabeculectomy. Poor methodology in the reviewed literature, along with bias meant that firm conclusions were difficult to draw; however, they were unable to exclude a beneficial effect from NPFS. Fewer complications occurred in the NPFS group compared to trabeculectomy (17% vs. 65%). The impact on quality-of-life has been recognized as a potential benefit of NPFS over filtering surgery, requiring further research [11]. DS has been found to induce less astigmatism than trabeculectomy, though it can still induce refractive changes [12]. We have previously undertaken a morphological comparison of trabeculectomy and DS blebs, with DS forming a fluid reservoir below the flap. This is opposed to trabeculectomy, where the reservoir forms above the scleral flap. We found that the height of the reservoir and subconjunctival bleb are associated with better outcomes [13].

Dwivedi et al. reported a retrospective review of both DS combined with MMC and trabeculectomy with MMC over two years of follow-up. They found a mean IOP reduction of 33.94% in the DS group and 38.39% in the trabeculectomy group. Most patients achieved an IOP of 16 mmHg or less (82.61% in the DS group, 95.46% in the trabeculectomy group). More patients in the trabeculectomy group achieved an IOP of 12 mmHg or under (52.17% vs. 72.72%), as well as a greater than 30% reduction in IOP (54.35% vs. 68.18%) [14].

Kozobolis et al. described a modification to DS in which the AC was penetrated (“DS plus trabeculectomy”) in eyes with POAG, or pseudoexfoliative glaucoma. Over a three-year follow-up period, they described an average reduction of 11.24 mmHg and a qualified success rate (IOP <22 mmHg with or without medication) of 75.86%. There was a low rate of complications in addition to a reduction in medication use [15].

Sanchez et al. compared eyes who underwent DS with subsequent TDW puncture to a comparative set of trabeculectomy eyes and found similar outcomes. They highlighted the importance of iridectomy in eyes with angle closure to ensure the area of sclerectomy is clear of the iris [9]; this may, however, incite inflammation. DS has traditionally been avoided in eyes with angle closure due to the possibility of the iris blocking the TDW. Yuen et al. found that combined DS and phacoemulsification can be a safe and effective surgical option for the management of these eyes. They found nearly 52% had a reduction in IOP without the use of any medication [16]. 5% of eyes in our group had a form of angle closure, and all underwent combined DS and phacoemulsification aside from one eye. In this eye (with a history of corneal graft and secondary angle closure), the angle was open in the superior region where surgery was undertaken.

DS may be combined with cataract surgery, which can be controversial when combined with trabeculectomy [17]. Botz and Heider reviewed the results of DS combined with cataract surgery in 300 eyes and found similar IOP reductions and complications to trabeculectomy alone [18]. Lochead et al. found better IOP reduction with combined DS and cataract surgery than trabeculectomy alone [19], as did Kleinmann et al. [20]. Gianoli et al. found similar results between DS and phacoemulsification compared with phacotrabeculectomy at one year [21]. The apparent benefit of combined phacoemulsification and DS over trabeculectomy has been postulated to be due to greater surgical trauma and breakdown of the blood-aqueous barrier in the latter. An increase in transforming growth factor, leading to a greater risk of trabeculectomy failure, has also been identified [22,23].

Overall, trabeculectomy has been found to have superior results with regards to IOP lowering, though DS performs better when combined with cataract surgery [24]. Deliseo et al. found that IOP control was better in those with combined surgery than DS alone (13.1 vs. 15.2 mmHg); 90% of this group achieved IOP under 21 mmHg without any glaucoma medication, compared to 62% of those with DS alone. Rates of hypotony were lower in those with combined surgery [25].

In our retrospective review, we found good results in terms of IOP-lowering following DS, with a mean IOP of 13.61 mmHg and a mean reduction of 42.21%. Pre-operative IOP and final IOP were slightly higher in eyes that did not have concurrent phacoemulsification than those that had both DS and phacoemulsification with IOL insertion (pre-operative 28.44 mmHg vs. 20.00 mmHg; final 14.21 mmHg vs. 13.17 mmHg); this is in concordance with the literature discussed above. IOP was lowered by a mean of 34.15% in the phacoemulsification group and 50.04% in eyes without phacoemulsification. There was a reduction in the number of anti-glaucoma medications. Perioperative complications were rare. Two eyes (2.5%) were converted to trabeculectomy during the procedure, with 8 eyes (10%) later undergoing YAG goniopuncture. These patients had a mean final IOP of 14.6 mmHg. As described above, we found a complete success rate of 67.5%, slightly lower than the published results in the literature. Considering further procedures subsequently performed in our population, we had a qualified success rate of 88.8% (though this includes those with subsequent trabeculectomy and GDD surgery).

Postoperative complications were fairly common. Numerical hypotony and raised IOP occurred in 17.5% and 25%, respectively, though they were usually transient. Hypotonous complications affecting vision (maculopathy, choroidal effusion, and AC shallowing) were rarer (1.25%; 7.5%; 1.25%, respectively). Of these, two patients required bleb revision. A review of 1765 eyes by Rabiolo et al. found chronic hypotony rates of 13.4% following DS with or without cataract extraction (IOP ≤5 mm Hg in ≥2 consecutive visits lasting >90 days), with clinical hypotony (visually significant complications) in 5.6% over five years. They recognized a large drop in IOP in the first week post-operatively, later rising to a more stable profile in the low-to-mid teens, similar to our patients, where the trend was to a lower IOP at the first post-operative assessment [26].

As is often the case following glaucoma surgery, we found a high rate of eyes requiring further procedures after their DS, including YAG goniopuncture (10%) and bleb needling (13.75%). Penetrating filtering surgery was required in 8.75% of eyes, with 3.75% undergoing GDD surgery. This highlights an important part of the consent process for DS.

There are several limitations to our review. This was a retrospective review undertaken as part of an internal audit process, and the long data collection period spanned the transition of our hospital from paper-based to electronic records; as such, some data was unavailable, particularly for those undertaken earlier. There was no randomization or comparison between different groups, which adds bias to the statistical analysis. There is also a wide range of follow-up times for different patients. This does, however, reflect real-world practice where follow-up can be limited due to patient attendance.

## Conclusions

We have found DS to be an effective method of reducing IOP, with good results in patients undergoing concurrent phacoemulsification and IOL insertion. It is a useful procedure for glaucoma surgeons to have in their toolkit, appearing to offer fewer complications than traditional penetrating surgery, albeit with a higher-end IOP. It is particularly useful in eyes where surgery such as trabeculectomy carries a risk of failure. High-quality comparative studies are limited, and future work involving randomized controlled trials against other common glaucoma procedures, including GDDs and micro-invasive glaucoma surgeries, as well as differences in quality-of-life measures, would be useful.

## References

[1] Roy S, Mermoud A (2017). Deep sclerectomy. Dev Ophthalmol.

[2] Arimura S, Iwasaki K, Orii Y, Takamura Y, Inatani M (2021). Comparison of 5-year outcomes between trabeculectomy combined with phacoemulsification and trabeculectomy followed by phacoemulsification: a retrospective cohort study. BMC Ophthalmol.

[3] Guedes RA, Guedes VM (2006). [Non-penetrating filtering surgery: concept, technique and results]. Arq Bras Oftalmol.

[4] Baudouin C, Hamard P, Labbé A (2007). Surgical key points. Nonpenetrating sclerectomy. J Fr Ophtalmol.

[5] Bylsma S (1999). Nonpenetrating deep sclerectomy: collagen implant and viscocanalostomy procedures. Int Ophthalmol Clin.

[6] Rebolleda G, Muñoz-Negrete FJ (2004). Phacoemulsification-deep sclerotomy converted to phacotrabeculectomy. J Cataract Refract Surg.

[7] Kałużny JJ, Grzanka D, Wiśniewska H, Niewińska A, Kałużny BJ, Grzanka A (2012). Intrascleral outflow after deep sclerectomy with absorbable and non-absorbable implants in the rabbit eye. Med Sci Monit.

[8] Tan JC, Hitchings RA (2001). Non-penetrating glaucoma surgery: the state of play. Br J Ophthalmol.

[9] Sanchez E, Schnyder CC, Mermoud A (1997). Comparative results of deep sclerectomy transformed to trabeculectomy and classical trabeculectomy. Klin Monbl Augenheilkd.

[10] Cheng JW, Cheng SW, Cai JP, Li Y, Wei RL (2011). Systematic overview of the efficacy of nonpenetrating glaucoma surgery in the treatment of open angle glaucoma. Med Sci Monit.

[11] Eldaly MA, Bunce C, Elsheikha OZ, Wormald R (2014). Non-penetrating filtration surgery versus trabeculectomy for open-angle glaucoma. Cochrane Database Syst Rev.

[12] Chan HH, Kong YX (2017). Glaucoma surgery and induced astigmatism: a systematic review. Eye Vis (Lond).

[13] Konstantopoulos A, Yadegarfar ME, Yadegarfar G, Stinghe A, Macleod A, Jacob A, Hossain P (2013). Deep sclerectomy versus trabeculectomy: a morphological study with anterior segment optical coherence tomography. Br J Ophthalmol.

[14] Dwivedi R, Somerville T, Cheeseman R, Rogers C, Batterbury M, Choudhary A (2021). Deep sclerectomy and trabeculectomy augmented with Mitomycin C: 2-year post-operative outcomes. Graefes Arch Clin Exp Ophthalmol.

[15] Kozobolis V, Kalogianni E, Sideroudi H (2020). Penetrating deep sclerectomy in primary open-angle and pseudoexfoliative glaucoma. Eur J Ophthalmol.

[16] Yuen NS, Chan OC, Hui SP, Ching RH (2007). Combined phacoemulsification and nonpenetrating deep sclerectomy in the treatment of chronic angle-closure glaucoma with cataract. Eur J Ophthalmol.

[17] Verges C, Cazal J, Lavin C (2005). Surgical strategies in patients with cataract and glaucoma. Curr Opin Ophthalmol.

[18] Botz N, Heider W (2004). Long-term influence of pre-, intra-, and postoperative factors on the intraocular pressure in combined cataract and glaucoma surgery. Ophthalmologe.

[19] Lochhead J, Casson RJ, Salmon JF (2003). Long term effect on intraocular pressure of phacotrabeculectomy compared to trabeculectomy. Br J Ophthalmol.

[20] Kleinmann G, Katz H, Pollack A, Schechtman E, Rachmiel R, Zalish M (2002). Comparison of trabeculectomy with mitomycin C with or without phacoemulsification and lens implantation. Ophthalmic Surg Lasers.

[21] Gianoli F, Schnyder CC, Bovey E (1999). Combined surgery for cataract and glaucoma: phacoemulsification and deep sclerectomy compared with phacoemulsification and trabeculectomy. J Cataract Refract Surg.

[22] Siriwardena D, Kotecha A, Minassian D, Dart JK, Khaw PT (2000). Anterior chamber flare after trabeculectomy and after phacoemulsification. Br J Ophthalmol.

[23] Vass C, Menapace R (2004). Surgical strategies in patients with combined cataract and glaucoma. Curr Opin Ophthalmol.

[24] Chiselita D (2001). Non-penetrating deep sclerectomy versus trabeculectomy in primary open-angle glaucoma surgery. Eye (Lond).

[25] D'Eliseo D, Pastena B, Longanesi L, Grisanti F, Negrini V (2003). Comparison of deep sclerectomy with implant and combined glaucoma surgery. Ophthalmologica.

[26] Rabiolo A, Leadbetter D, Anand N (2021). Hypotony-associated complications after deep sclerectomy: incidence, risk factors, and long-term outcomes. J Glaucoma.

